# β-Amino functionalization of cinnamic Weinreb amides in ionic liquid

**DOI:** 10.3762/bjoc.12.231

**Published:** 2016-11-11

**Authors:** Yi-Ning Wang, Guo-Xiang Sun, Gang Qi

**Affiliations:** 1 School of Chemistry & Chemical Engineering, Yancheng Institute of Technology, Yancheng, Jiangsu 224051, China

**Keywords:** aminochlorination, β-amino functionalization, ionic liquid, Weinreb amides

## Abstract

2-Ns-Protected β-amino Weinreb amides were synthesized by aminochlorination of α,β-unsaturated Weinreb amides in an ionic liquid, 1-*n*-butyl-3-methylimidazolium bis(trifluoromethanesulfonyl)imide ([BMIM][NTf_2_]). Processed without the use of metal catalysts or the need of an inert gas atmosphere, the presented process can be readily performed as a one-pot synthesis at room temperature. Moreover, the preparation has the distinct advantages of the use of 2-NsNCl_2_ as an inexpensive and stable nitrogen/halogen source and the ionic liquid as a recyclable reaction media. Nine examples were examined, and modest to good isolated chemical yields (40–83%) were obtained.

## Introduction

Unlike *N*-protected α-amino carbonyl compounds, their analogues, the β-amino carbonyl compounds, have drawn relatively little attention. However, the β-amino carbonyl moieties are not only found in natural products [1–3], e.g., in the side chain of Taxol, but also in important building blocks widely used in the organic syntheses. They are especially used in the synthesis of β-amino acids [4–5], which are precursors for many biological and pharmacological active compounds, such as β-lactams [6] and β-peptides [7]. The most straightforward approach towards the preparation of β-amino carbonyl compounds is the Mannich reaction [8–9]. However, there are few reported methods specifically focused on this target, which include oxidation of γ-amino alcohols [10], hydrolysis of 1,3-oxazines [11], rearrangement of 2,3-aziridinio alcohols [12], etc.

**Scheme 1:**
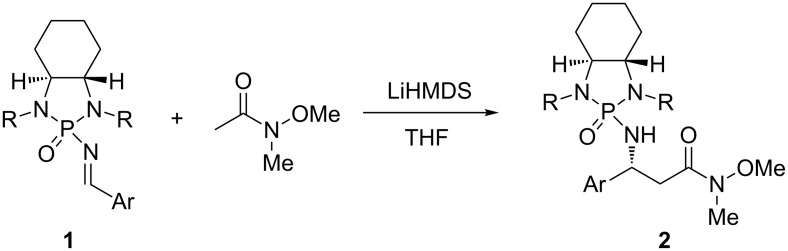
Synthesis of *N*-phosphonyl-β-amino Weinreb amides.

*N*-Methoxy-*N*-methylamides, known as Weinreb amides, were first reported by Nahm and Weinreb in 1981 [13]. Since then, the Weinreb amides were found to be useful precursors for the synthesis of their carbonyl equivalents in organic chemistry [14]. By treating with various nucleophiles, such as hydrides, Grignard and organolithium reagents or ester enolates, the Weinreb amides can be easily converted into aldehydes, ketones, and β-keto esters, respectively [13–20]. Therefore, the transformation of β-amino Weinreb amides can provide a promising pathway towards the achievement of β-amino carbonyl derivatives. Surprisingly, there have been only a few successful preparations for β-amino Weinreb amides to date. One of which is the addition of the enolate of commercially available *N*-methoxy-*N*-methylacetamide to sulfinimines to generate the corresponding *N*-sulfinyl β-amino Weinreb amides [21–26]. Another approach was the direct amination of α,β-unsaturated Weinreb amides by using lithium (*S*)-*N*-benzyl-*N*-α-methylbenzylamide as the nitrogen source [27–29]. Recently, a new chiral *N*-phosphonylimine chemistry was developed by the Li group. By reacting *N*-phosphonylimines **1** with Weinreb amides, *N*-phosphonyl-β-amino Weinreb amides **2** can be obtained (Scheme 1) [30]. Although great progress has already been achieved, there were still some limitations in the existing syntheses of β-amino Weinreb amides, such as the difficult syntheses of starting materials or the harsh reaction conditions required for the removal of the *N*-protecting groups.

**Scheme 2:**
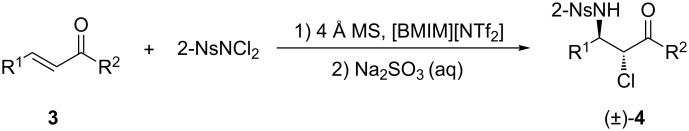
Aminochlorination of α,β-unsaturated ketones in ionic liquid.

With the aminochlorination developed in the past few years [31], we found that when the aminochlorination reactions of α,β-unsaturated ketones **3** were carried out in ionic liquid, [BMIM][NTf_2_] (1-*n*-butyl-3-methylimidazolium bis(trifluoromethanesulfonyl)imide, Tf = SO_2_CF_3_), a series of α-chloro-β-amino ketone derivatives **4** could be obtained with excellent regioselectivities (Scheme 2) [32]. Encouraged by these promising results, we thought that the α,β-unsaturated Weinreb amides could be potential substrates for the preparation of β-amino Weinreb amides. Compared with the reported methods, there are several advantages for the proposed aminochlorination reactions: 1) it is much easier to achieve the starting materials, including the α,β-unsaturated Weinreb amides and the nitrogen sources, such as 4-TsNCl_2_ and 2-NsNCl_2_; 2) the removal of the *N*-protecting groups is relatively easier than those shown above; and 3) the ionic liquids are environmentally friendly and can be readily recycled.

## Results and Discussion

The first attempt was to conduct the aminochlorination reaction of *N*-methoxy-*N*-methylcinnamoylamide (**5a**) in acetonitrile by using 2-NsNCl_2_ as the nitrogen source and Cu(I)OTf as the metal catalyst [33–34]. However, there no aminochlorination products were observed. Next, we turned our attention to the use of ionic liquids, which have shown many significant advantages in the reported aminochlorination reactions [35–37]. However, when the ionic liquid [BMIM][BF_4_] was utilized as the reaction media, no anticipated haloamine products were detected, which was similar to the aminochlorination of α,β-unsaturated ketones [32]. Fortunately, when we switched the ionic liquid to [BMIM][NTf_2_], it was found that the starting material, *N*-methoxy-*N*-methylcinnamoylamide (**5a**), was consumed completely after 24 h without utilizing any metal catalysts. Furthermore, under the optimized reaction conditions, the isolated chemical yield of the β-amino product could be increased up to 81% (Table 1). A series of common α,β-unsaturated Weinreb amides were systematically investigated as well. Similarly, the aminochlorination reactions proceeded smoothly, and the anticipated α-chloro-β-amino products were obtained in modest to good yields and excellent regioselectivities (Table 2).

**Table 1:** Optimization of aminochlorination of *N*-methoxy-*N*-methylcinnamoylamide (**5a**).

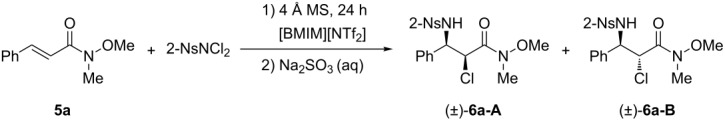			

Entry	Temp (°C)	Cu(I)OTf	Yield (%)^a^

1	25	–	81
2	25	10 mol %	71
3	80	–	60
4	80	10 mol %	61

^a^Combined yields of two isomers purified by column chromatography.

**Table 2:** Results of aminochlorination of α,β-unsaturated Weinreb amides.

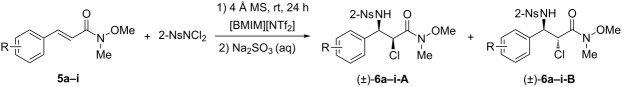					

Entry	Substrate	R	Product	Yield^a^ (%)	Stereoselectivity^b^ (**A**:**B**)

1	**5a**	H	**6a**	81	1.0:1.3
2	**5b**	2-fluoro	**6b**	83	1.0:1.5
3	**5c**	4-chloro	**6c**	70	1.0:1.4
4	**5d**	2,4-dichloro	**6d**	79	1.0:1.8
5	**5e**	4-bromo	**6e**	79	1.0:1.3
6	**5f**	4-methyl	**6f**	78	1.0:1.2
7	**5g**	4-methoxy	**6g**	63	1.0:1.2
8	**5h**	4-phenyl	**6h**	69	1.0:0.7
9	**5i**	4-naphthyl	**6i**	40	1.0:0.7

^a^Combined yields of two isomers separated by column chromatography. ^b^Determined after column chromatography.

**Scheme 3:**
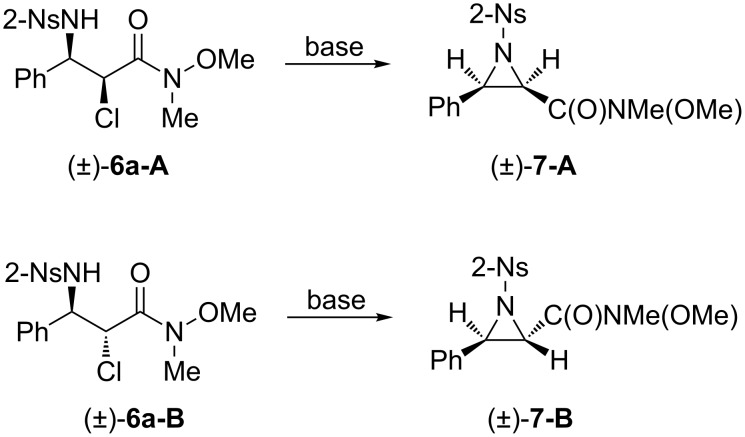
Conversion of β-amino Weinreb amides to aziridines.

Different from the similar aminochlorination reactions of α,β-unsaturated ketones mediated in the same ionic liquid, two different isomers were formed nearly equally. However, these diastereomers can be easily separated by flash column chromatography. The regioselectivities of the isomers were determined by mass spectrometric analyses, both of which prominent peaks corresponding to [PhCHNHNs]^+^ (*m*/*z* = 291) could be clearly observed. The results indicated that the isolated products were both α-chloro-β-amino Weinreb amide derivatives. The assignments for the stereoselectivities were determined by the conversions of the diastereomers to their corresponding aziridines (Scheme 3), and diastereomers **6a-A** and **6a-B** were chosen as representative substrates. Based on the vicinal Karplus correlation diagram, the vicinal proton coupling constants in the ^1^H NMR spectra of *cis*-aziridines are larger than those of *trans*-azridines [38–39]. In the ^1^H NMR spectra of the resulting aziridines derived from compounds **6a-A** and **6a-B**, the vicinal proton coupling constants were 7.5 Hz and 4.5 Hz, respectively. Therefore, compound **6a-A** was assigned as *syn*-stereoisomer, and compound **6a-B** was assigned as *anti*-stereoisomer.

**Figure 1:**
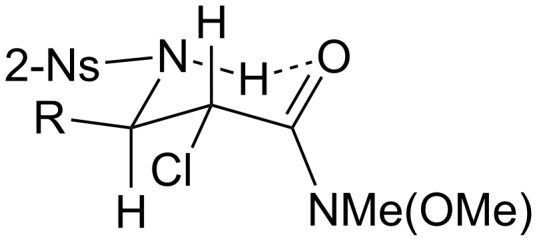
Possible hydrogen bonding conformation.

**Scheme 4:**
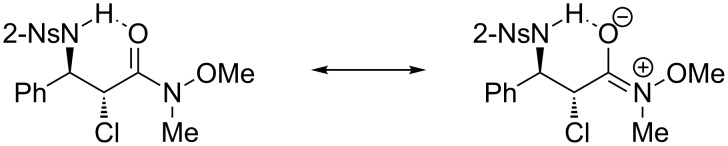
Resonance structures of Weinreb amides.

**Scheme 5:**
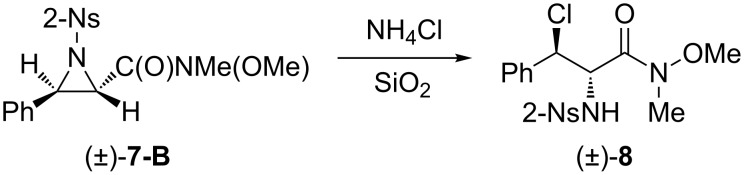
Transformation of *trans*-aziridine.

In the ^1^H NMR spectra of the isolated diastereomers, an obvious and interesting phenomenon can be seen; namely, the difference of the proton chemical shifts of the hydrogen atoms of the nitrobenzenesulfonylamines. Compared with the chemical shifts of these protons belonging to the *syn*-stereoisomers, the signals of the same kind of protons of the *anti*-stereoisomers move to much lower fields (e.g., 8.25 ppm vs 6.33 ppm for compounds **6a-B** vs **6a-A**). The reason for this phenomenon is probably due to hydrogen bonding between the protons of the nitrobenzenesulfonyl amines and the neighboring carbonyl groups, which form stable six-membered rings as shown in Figure 1. In addition, because of the resonance structures (Scheme 4), the hydrogen bonding can be further strengthened by the oxygen which is more electronegative. As a result, the chemical shifts of these protons belonging to the *anti*-stereoisomers can move to much lower fields than those of the similar hydrogen atoms of not only the *syn*-stereoisomers but also the β-amino compounds derived from α,β-unsaturated ketones. This hypothesis can be supported by an unexpected transformation. When aziridine **7-B** was purified by flash column chromatography, part of the aziridine ring was opened by a chlorine anion to form the α-amino-β-chloro Weinreb amide derivative **8** (Scheme 5). This could be confirmed by mass spectroscopic analysis as well. In its mass spectrum, a prominent peak denoting [NsNHCHC(O)NMe(OMe)]^+^ (*m*/*z* = 302) was exclusively observed. Because compound **8** was derived from *trans*-aziridine, it should be the *trans*-regioisomer of compound **6a-B**. As expected, its proton chemical shift of nitrobenzenesulfonylamine was only 6.20 ppm.

**Scheme 6:**
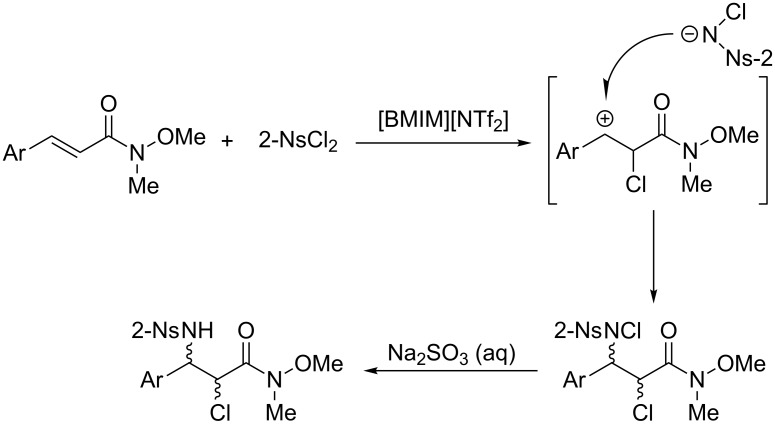
Proposed mechanism of aminochlorination of α,β-unsaturated Weinreb amides.

As shown in Table 2, nearly 1:1 stereoselectivities were observed in all cases. This indicated that instead of a chlorinium or aziridinium ion intermediate, as hypothesized in the previous analogous reactions, a relatively stable carbocation intermediate was more likely to have formed in this aminochlorination reaction (Scheme 6). Moreover, when the carbocation intermediate was formed, strong electron-withdrawing groups at the phenyl ring in the 3-position of the carbocation are highly disfavored for the aminochlorination reaction. For example, when the nitro group was attached to the phenyl ring, no aminochlorination reaction took place, and only starting material remained even after stirring for a long time.

## Conclusion

A new approach to afford β-amino Weinreb amides was achieved by the aminochlorination of α,β-unsaturated Weinreb amides. The new process has the advantages that the starting materials can be easily achieved and the *N*-protecting groups simply removed. Additionally, the reactions can be readily performed in the ionic liquid [BMIM][NTf_2_] at room temperature without the use of metal catalysts or the need for inert gases protection, and the final products can be easily isolated by flash column chromatography. Compared with the existing methods, the aminochlorination reactions of α,β-unsaturated Weinreb amides provided a promising direction towards the preparation of β-amino Weinreb amides, which have shown great importance in not only organic synthesis, but also in medicinal and pharmaceutical chemistry.

## Experimental

**General procedure:** α,β-Unsaturated Weinreb amides were prepared following literature methods [40]. The ionic liquid [BMIM][NTf_2_] was readily prepared by reacting 1-methylimidazole with *n*-butyl bromide [41–43], followed by anion metathesis using *N*-lithiotrifluoromethanesulfonimide in acetone solution. The resulting ionic liquid, [BMIM][NTf_2_], was carefully dried by heating at 60 °C in vacuum, then confirmed by ^1^H NMR analysis [44]. All aminochlorination reactions were performed in oven-dried vials. Flash column chromatography was performed using silica gel (Merck 60, 230–400 mesh). ^1^H and ^13^C NMR spectra were obtained with Varian Inova 500 MHz and Varian Mercury Plus 300 MHz spectrometers using deuterated chloroform as a solvent. Internal TMS (δ = 0.0 ppm) was used as the reference for ^1^H NMR, while the deuterated chloroform peak (δ = 77.0 ppm) was used as the reference for ^13^C NMR.

**Typical procedure for the aminochlorination of α,β-unsaturated Weinreb amides 5a**–**i**: Analogous to the procedure described in [32], *N*-methoxy-*N*-methylcinnamoylamide (**5a**, 96 mg, 0.5 mmol, 1.0 equiv), 4 Å molecular sieves (100 mg), 2-NsNCl_2_ (163 mg, 0.6 mmol, 1.2 equiv) and [BMIM][NTf_2_] (500 mg) were loaded into an oven-dried vial. The resulting mixture was stirred at room temperature for 24 h. The reaction was finally quenched with saturated aqueous Na_2_SO_3_solution. The product was extracted with ethyl acetate (5 mL × 3) and the combined organic phases were washed with brine and dried over anhydrous Na_2_SO_4_. The crude product was subjected to flash column chromatography (EtOAc/dichloromethane/hexane, v/v/v = 1:3:2) to yield 174 mg of product as a white solid (81%).

**Typical procedure for the preparation of aziridines 7A and 7B:** The β-amino Weinreb amide (**6a-A** or **6a-B**, 70 mg, 0.16 mmol, 1.0 equiv), dichloromethane (5 mL) and triethylamine (0.1 mL) were loaded into an oven-dried vial. After stirring at room temperature for 24 h, the reaction mixture was concentrated to dryness and purified by flash column chromatography (EtOAc/hexane, v/v = 1:3) to yield the product as a white solid.

## Supporting Information

File 1 Full compound characterization data for products **6a**–**i, 7A, 7B** and **8**.
