# Supplementary material for: β-Amino functionalization of cinnamic Weinreb amides in ionic liquid
Source: Beilstein J Org Chem. 2016 Nov 11;12:2372–7. doi: 10.3762/bjoc.12.231 (PMC5238596; doi:10.3762/bjoc.12.231)
Supplement: File 1 — Full compound characterization data for products 6a–i, 7A, 7B and 8. [file Beilstein_J_Org_Chem-12-2372-s001.pdf]

# Supporting Information

**for**

# β-Amino functionalization of cinnamic Weinreb amides in ionic liquid

Yi-Ning Wang*, Guo-Xiang Sun and Gang Qi

Address: School of Chemistry & Chemical Engineering, Yancheng Institute of Technology, Yancheng, Jiangsu 224051, China

Email: Yi-Ning Wang - yining.wang@ycit.cn

*Corresponding author

## Full compound characterization data for products 6a–i, 7A, 7B and 8

## Characterisation of products:

**6a-A**: Isolated as white solid; $^1$H NMR (500 MHz, $CDCl_3$): δ = 7.85-7.83 (dd, J = 1.5, 8.0 Hz,1 H), 7.80-7.78 (dd, J = 1.5, 8.0 Hz, 1 H), 7.62-7.59 (m, 1 H), 7.55-7.51 (m, 1 H), 7.25-7.23 (m, 2 H), 7.18-7.15 (m, 3 H), 6.33-6.31 (d, J = 7.0 Hz, 1 H), 5.10-5.07 (t, 1 H), 5.04-5.02 (d, J = 8.5 Hz, 1 H), 3.57 (s, 3 H), 2.99 (s, 3 H) ppm; $^{13}$C NMR (125 MHz, $CDCl_3$): δ = 166.4, 147.4, 136.2, 134.1, 133.2, 132.7, 130.9, 128.7, 128.5, 127.7, 125.2, 61.7, 60.7, 55.8, 32.2 ppm. **6a-B**: Isolated as white solid; $^1$H NMR (500 MHz, $CDCl_3$): δ = 8.26-8.24 (d, J = 8.5 Hz, 1 H), 7.80-7.78 (dd, J = 1.0, 8.0 Hz,1 H), 7.76-7.74 (dd, J = 1.5, 8.0 Hz, 1 H), 7.58-7.55 (m, 1 H), 7.45-7.42 (m, 1 H), 7.26-7.23 (m, 2 H), 7.18-7.15 (m, 3 H), 5.11-5.08 (dd, J = 4.5, 8.5 Hz, 1 H), 5.02-5.01 (d, J = 4.5 Hz, 1 H), 3.36 (s, 3 H), 3.09 (s, 3 H) ppm; $^{13}$C NMR (125 MHz, $CDCl_3$): δ = 167.7, 147.5, 136.4, 135.2, 132.8, 132.3, 130.2, 128.6, 128.5, 127.1, 124.9, 62.1, 61.6, 51.1, 32.0 ppm.

**6b-A**: Isolated as white solid; $^1$H NMR (500 MHz, $CDCl_3$): δ = 7.95-7.93 (dd, J = 1.5, 8.0 Hz,1 H), 7.80-7.78 (dd, J = 1.5, 8.0 Hz, 1 H), 7.62-7.55 (m, 2 H), 7.28-7.24 (m, 1 H), 7.19-7.14 (m, 1 H), 6.97-6.94 (m, 1 H), 6.89-6.85 (m, 1 H), 6.44-6.42 (d, J = 9.0 Hz, 1 H), 5.31-5.28 (t, 1 H), 5.22-5.21 (d, J = 8.5 Hz, 1 H), 3.68 (s, 3 H), 2.98 (s, 3 H) ppm; $^{13}$C NMR (125 MHz, $CDCl_3$): δ = 166.5, 161.3, 159.3, 147.3, 134.3, 133.3, 132.7, 130.8, 130.7, 125.3, 124.3, 124.2, 115.7, 115.5, 61.9, 57.3, 54.7, 32.3 ppm. **6b-B**: Isolated as white solid; $^1$H NMR (500 MHz, $CDCl_3$): δ = 8.03-8.01 (d, J = 9.0 Hz, 1 H), 7.91-7.89 (dd, J = 1.50, 8.0 Hz,1 H), 7.83-7.81 (dd, J = 1.0, 8.0 Hz, 1 H), 7.65-7.62 (m, 1 H), 7.57-7.54 (m, 1 H), 7.37-7.34 (m, 1 H), 7.23-7.19 (m, 1 H), 6.99-6.95 (m, 1 H), 5.39-5.36 (dd, J = 5.0,

9.0 Hz, 1 H), 5.12-5.11 (d, J = 5.0 Hz, 1 H), 3.50 (s, 3 H), 3.07 (s, 3 H) ppm; $^{13}C$ NMR (125 MHz, $CDCl_3$): δ = 167.5, 160.6, 158.7, 147.3, 134.5, 133.2, 132.5, 130.4, 130.3, 125.0, 124.4, 124.3, 123.5, 115.3, 115.1, 61.7, 56.1, 50.0, 31.9 ppm.

**6c-A**: Isolated as white solid; $^{1}H$ NMR (500 MHz, $CDCl_3$): δ = 7.85-7.82 (m, 2 H), 7.68-7.65 (m, 1 H), 7.60-7.57 (m, 1 H), 7.24-7.21 (m, 2 H), 7.18-7.15 (m, 2 H), 6.37-6.35 (d, J = 6.5 Hz, 1 H), 5.05-5.02 (dd, J = 7.0, 8.5 Hz,1 H), 5.00-4.98 (d, J = 9.0 Hz, 1 H), 3.61 (s, 3 H), 3.01 (s, 3 H) ppm; $^{13}C$ NMR (125 MHz, $CDCl_3$): δ = 166.2, 147.4, 135.0, 134.6, 133.9, 133.4, 132.8, 130.8, 129.2, 128.7, 125.3, 61.8, 60.1, 55.3, 32.3 ppm. **6c-B**: Isolated as white solid; $^{1}H$ NMR (500 MHz, $CDCl_3$): δ = 8.22-8.21 (d, J = 8.5 Hz, 1 H), 7.82-7.81 (dd, J = 1.0, 8.0 Hz,1 H), 7.79-7.77 (dd, J = 1.0, 8.0 Hz, 1 H), 7.64-7.60 (m, 1 H), 7.52-7.48 (m, 1 H), 7.24-7.21 (m, 2 H), 7.20-7.14 (m, 2 H), 5.09-5.06 (dd, J = 5.0, 8.5 Hz, 1 H), 5.00-4.99 (d, J = 5.0 Hz, 1 H), 3.48 (s, 3 H), 3.10 (s, 3 H) ppm; $^{13}C$ NMR (125 MHz, $CDCl_3$): δ = 167.5, 147.5, 135.2, 134.9, 134.5, 133.0, 132.4, 130.2, 128.8, 128.7, 125.0, 61.8, 61.5, 51.0, 32.0 ppm.

**6d-A**: Isolated as white solid; $^{1}H$ NMR (500 MHz, $CDCl_3$): δ = 7.91-7.86 (m, 2 H), 7.69-7.65 (m, 1 H), 7.61-7.58 (m, 1 H), 7.43-7.41 (d, J = 8.5 Hz, 1 H), 7.26-7.25 (d, J = 2.0 Hz, 1 H), 7.14-7.12 (dd, J = 2.0, 8.5 Hz, 1 H), 6.69 (s, 1 H), 5.48-5.47 (d, J = 6.5, 1 H), 5.28-5.27 (d, J = 6.0 Hz, 1 H), 3.72 (s, 3 H), 3.04 (s, 3 H) ppm; $^{13}C$ NMR (125 MHz, $CDCl_3$): δ = 166.3, 147.4, 135.2, 134.1, 133.4, 133.1, 132.8, 132.7, 132.1, 130.5, 129.6, 127.2, 125.4, 62.0, 57.4, 54.8, 32.4 ppm. **6d-B**: Isolated as white solid; $^{1}H$ NMR (500 MHz, $CDCl_3$): δ = 8.32-8.30 (d, J = 8.5 Hz,

1 H), 7.95-7.93 (dd, J = 1.5, 8.0 Hz,1 H), 7.87-7.85 (dd, J = 1.5, 8.0 Hz, 1 H), 7.68-7.65 (m, 1 H), 7.62-7.59 (m, 1 H), 7.46-7.44 (d, J = 8.5 Hz, 1 H), 7.34-7.33 (d, J = 2.5 Hz, 1 H), 7.12-7.10 (dd, J = 2.0, 8.5 Hz, 1 H), 5.42-5.39 (dd, J = 4.5, 8.5 Hz, 1 H), 5.16-5.15 (d, J = 4.5 Hz, 1 H), 3.49 (s, 3 H), 3.07 (s, 3 H) ppm; $^{13}$C NMR (125 MHz, $CDCl_3$): δ = 167.4, 147.5, 135.2, 134.7, 133.2, 133.1, 132.9, 132.6, 130.5, 130.2, 129.3, 127.6, 125.2, 61.8, 58.6, 48.8, 32.0 ppm.

**6e-A**: Isolated as white solid; $^{1}$H NMR (500 MHz, $CDCl_3$): δ = 7.84-7.82 (dd, J = 1.5, 8.0 Hz, 2 H), 7.68-7.65 (m, 1 H), 7.60-7.56 (m, 1 H), 7.33-7.30 (m, 2 H), 7.18-7.15 (m, 2 H), 6.37-6.35 (d, J = 6.5 Hz, 1 H), 5.04-5.01 (dd, J = 7.0, 8.5 Hz,1 H), 5.00-4.98 (d, J = 9.0 Hz, 1 H), 3.61 (s, 3 H), 3.02 (s, 3 H) ppm; $^{13}$C NMR (125 MHz, $CDCl_3$): δ = 166.2, 147.4, 135.5, 133.9, 133.4, 132.8, 131.6, 130.8, 129.5, 125.3, 122.8, 61.8, 60.1, 55.2, 32.3 ppm. **6e-B**: Isolated as white solid; $^{1}$H NMR (500 MHz, $CDCl_3$): δ = 8.23-8.21 (d, J = 8.5 Hz, 1 H), 7.82-7.80 (dd, J = 1.0, 8.0 Hz,1 H), 7.78-7.76 (dd, J = 1.0, 8.0 Hz, 1 H), 7.64-7.61 (m, 1 H), 7.52-7.49 (m, 1 H), 7.32-7.30 (m, 2 H), 7.17-7.15 (m, 2 H), 5.07-5.04 (dd, J = 4.5, 8.5 Hz, 1 H), 5.01-5.00 (d, J = 4.5 Hz, 1 H), 3.48 (s, 3 H), 3.10 (s, 3 H) ppm; $^{13}$C NMR (125 MHz, $CDCl_3$): δ = 167.5, 147.4, 135.6, 134.8, 133.0, 132.4, 131.7, 130.2, 128.9, 125.0, 122.6, 61.8, 61.5, 50.9, 32.0 ppm.

**6f-A**: Isolated as white solid; $^{1}$H NMR (500 MHz, $CDCl_3$): δ = 7.85-7.83 (dd, J = 1.5, 8.0 Hz, 1 H), 7.80-7.78 (dd, J = 1.5, 8.0 Hz, 1 H), 7.63-7.59 (m, 1 H), 7.54-7.51 (m, 1 H), 7.12-7.11 (d, J = 8.0 Hz, 2 H), 6.96-6.95 (d, J = 8.0 Hz, 2 H), 6.29-6.28 (d, J = 7.0 Hz, 1 H), 5.06-5.01 (m, 2 H), 3.57 (s, 3 H), 3.00 (s, 3 H), 2.23 (s, 3 H) ppm; $^{13}$C NMR (125 MHz, $CDCl_3$): δ = 166.5, 147.4, 138.5, 134.2,

133.2, 133.1, 132.6, 130.9, 129.1, 127.5, 125.2, 61.8, 60.5, 55.7, 32.2, 21.1 ppm. **6f-B**: Isolated as white solid; $^1$H NMR (500 MHz, $CDCl_3$): δ = 8.20-8.18 (d, J = 9.0 Hz, 1 H), 7.80-7.78 (dd, J = 1.5, 8.0 Hz,1 H), 7.76-7.74 (dd, J = 1.0, 8.0 Hz, 1 H), 7.58-7.55 (m, 1 H), 7.45-7.42 (m, 1 H), 7.13-7.12 (d, J = 8.0 Hz, 2 H), 6.96-6.95 (d, J = 8.0 Hz, 2 H), 5.07-5.04 (dd, J = 4.5, 8.5 Hz, 1 H), 5.00-4.99 (d, J = 4.5 Hz, 1 H), 3.39 (s, 3 H), 3.10 (s, 3 H) 2.23 (s, 3 H) ppm; $^{13}$C NMR (125 MHz, $CDCl_3$): δ = 167.8, 147.5, 138.3, 135.2, 133.5, 132.6, 132.2, 130.3, 129.3, 127.1, 124.9, 61.9, 61.6, 51.2, 32.0, 21.0 ppm.

**6g-A**: Isolated as white solid; $^1$H NMR (500 MHz, $CDCl_3$): δ = 7.85-7.82 (m, 2 H), 7.66-7.63 (m, 1 H), 7.59-7.55 (m, 1 H), 7.26-7.22 (m, 2 H), 7.19-7.17 (dd, J = 2.5, 8.0 Hz, 1 H), 6.76-6.74 (d, J = 8.5 Hz, 2 H), 6.30-6.29 (t, 1 H), 4.99-4.98 (d, J = 4.0 Hz, 2 H), 3.84 (s, 3 H), 3.63 (s, 3 H), 3.03 (s, 3 H) ppm; $^{13}$C NMR (125 MHz, $CDCl_3$): δ = 166.2, 155.0, 147.4, 133.9, 133.4, 132.6, 130.8, 129.5, 129.3, 127.5, 125.3, 122.4, 111.7, 61.8, 59.8, 56.1, 55.2, 32.3 ppm. **6g-B**: Isolated as white solid; $^1$H NMR (500 MHz, $CDCl_3$): δ = 8.16-8.14 (d, J = 8.5 Hz, 1 H), 7.83-7.81 (dd, J = 1.0, 8.0 Hz,1 H), 7.78-7.77 (dd, J = 1.0, 8.0 Hz, 1 H), 7.61-7.58 (m, 1 H), 7.50-7.47 (m, 1 H), 7.26-7.22 (m, 2 H), 7.19-7.16 (dd, J = 2.5, 8.0 Hz, 1 H), 6.75-6.73 (d, J = 9.0 Hz, 2 H), 5.03-5.00 (dd, J = 4.5, 8.5 Hz, 1 H), 4.99-4.98 (d, J = 4.5 Hz, 1 H), 3.83 (s, 3 H), 3.51 (s, 3 H), 3.12 (s, 3 H) ppm; $^{13}$C NMR (125 MHz, $CDCl_3$): δ = 167.6, 155.0, 147.5, 134.9, 133.0, 132.2, 130.2, 129.6, 129.1, 126.9, 125.0, 122.6, 111.8, 61.8, 61.1, 56.2, 51.1, 32.1 ppm.

**6h-A:** Isolated as white solid; $^1$H NMR (500 MHz, $CDCl_3$): δ = 7.84-7.83 (dd, *J* = 1.0, 8.0 Hz, 1 H), 7.80-7.78 (dd, *J* = 1.0, 8.0 Hz, 1 H), 7.59-7.56 (m, 1 H),

7.51-7.46 (m, 3 H), 7.44-7.41 (m, 2 H), 7.39-7.33 (m, 3 H), 7.32-7.30 (m, 2 H), 6.37-6.36 (d, *J* = 7.5 Hz, 1 H), 5.15-5.12 (t, 1 H), 5.09-5.07 (d, *J* = 9.0 Hz, 1 H), 3.61 (s, 3 H), 3.02 (s, 3 H) ppm; $^{13}$C NMR (125 MHz, $CDCl_3$): δ = 166.4, 147.3, 141.4, 140.0, 135.2, 134.2, 133.1, 132.7, 130.9, 129.7, 128.9, 128.2, 127.7, 127.1, 126.9, 125.2, 61.8, 60.5, 55.5, 32.3 ppm. **6h-B:** Isolated as white solid; $^{1}$H NMR (500 MHz, $CDCl_3$): δ = 8.26-8.24 (d, *J* = 9.0 Hz, 1 H), 7.81-7.79 (dd, *J* = 1.5, 8.0 Hz,1 H), 7.76-7.75 (dd, *J* = 1.5, 8.0 Hz, 1 H), 7.56-7.53 (m, 1 H), 7.48-7.46 (m, 2 H), 7.44-7.34 (m, 6 H), 7.33-7.31 (m, 2 H), 5.15-5.13 (dd, *J* = 4.5, 8.5 Hz, 1 H), 5.07-5.06 (d, *J* = 5.0 Hz, 1 H), 3.45 (s, 3 H), 3.12 (s, 3 H) ppm; $^{13}$C NMR (125 MHz, $CDCl_3$): δ = 167.8, 147.5, 141.3, 140.0, 135.4, 135.1, 132.7, 132.2, 130.4, 128.9, 127.7, 127.2, 126.9, 124.9, 61.9, 61.7, 51.2, 32.0 ppm.

**6i-A**: Isolated as white solid; $^{1}$H NMR (500 MHz, $CDCl_3$): δ = 8.22-8.20 (d, J = 8.5 Hz, 1 H),7.72-7.71 (d, J = 8.0 Hz, 1 H), 7.63-7.60 (m, 2 H), 7.56-7.53 (m, 1 H), 7.49-7.44 (m, 3 H), 7.39-7.35 (m, 1 H), 7.25-7.23 (d, J = 7.0 Hz, 1 H), 7.15-7.11 (m, 1 H), 6.49-6.48 (d, J = 8.5 Hz, 1 H), 6.00-5.97 (t, 1 H), 5.41-5.40 (d, J = 8.0 Hz, 1 H), 3.66 (s, 3 H), 2.96 (s, 3 H) ppm; $^{13}$C NMR (125 MHz, $CDCl_3$): δ = 166.8, 146.8, 134.0, 133.6, 132.8, 132.1, 130.5, 130.3, 129.3, 128.7, 126.8, 126.0, 125.7, 124.9, 124.7, 122.8, 61.8, 56.3, 32.4 ppm. **6i-B**: Isolated as white solid; $^{1}$H NMR (500 MHz, $CDCl_3$): δ = 8.46-8.44 (d, J = 8.0 Hz, 1 H), 8.02-8.00 (d, J = 8.5 Hz, 1 H), 7.83-7.82 (d, J = 8.0 Hz,1 H), 7.79-7.77 (dd, J = 1.5, 8.0 Hz, 1 H), 7.69-7.68 (d, J = 8.0 Hz, 1 H), 7.67-7.61 (m, 2 H), 7.58-7.57 (d, J = 7.5 Hz, 1 H), 7.54-7.50 (m, 2 H), 7.35-7.32 (m, 1 H), 7.24-7.21 (t, 1 H), 5.95-5.9 3 (dd, J = 4.5, 8.5 Hz, 1 H), 5.26-5.25 (d, J = 4.5 Hz, 1 H), 3.03 (s, 3 H), 2.96 (s, 3 H) ppm; $^{13}$C NMR (125 MHz, $CDCl_3$): δ = 167.7, 147.4, 134.9, 133.5, 132.8, 132.2, 131.5,

130.1, 130.0, 129.2, 129.1, 127.3, 126.0, 125.7, 125.1, 124.9, 121.4, 61.3, 58.1, 49.9, 31.9 ppm.

**7-A:** Isolated as white solid; $^{1}$H NMR (500 MHz, $CDCl_3$): δ = 8.50-8.48 (m, 1 H), 7.82-7.78 (m, 3 H), 7.41-7.39 (m, 2 H), 7.33-7.29 (m, 3 H), 4.43-4.42 (d, *J* = 7.5 Hz, 1 H), 4.33-4.31 (d, *J* = 7.5 Hz, 1 H), 3.66 (s, 3 H), 3.03 (s, 3H) ppm; $^{13}$C NMR (125 MHz, $CDCl_3$): δ = 163.6, 148.4, 134.6, 132.7, 132.2, 131.9, 131.6, 128.7, 128.4, 127.6, 124.7, 61.8, 47.3, 46.7, 32.4 ppm.

**7-B:** Isolated as white solid; $^{1}$H NMR (500 MHz, $CDCl_3$): δ = 8.21-8.19 (m, 1 H), 7.79-7.77 (m, 1 H), 7.72-7.70 (m, 2 H), 7.37-7.33 (m, 5 H), 4.64-4.63 (d, *J* = 4.5 Hz, 1 H), 4.21-4.20 (d, *J* = 4.5 Hz, 1 H), 3.92 (s, 3 H), 3.34 (s, 3H) ppm; $^{13}$C NMR (125 MHz, $CDCl_3$): δ = 164.7, 133.9, 133.1, 132.4, 130.6, 129.0, 128.6, 128.5, 128.1, 127.4, 124.8, 62.2, 49.9, 46.7, 32.8 ppm.

**8:** Isolated as white solid; $^{1}$H NMR (500 MHz, $CDCl_3$): δ = 7.83-7.81 (dd, *J* = 1.5, 8.0 Hz,1 H), 7.81-7.79 (dd, *J* = 1.5, 8.0 Hz, 1 H), 7.65-7.62 (m, 1 H), 7.60-7.56 (m, 1 H), 7.32-7.30 (m, 2 H), 7.21-7.16 (m, 3 H), 6.18-6.16 (d, *J* = 10.0 Hz, 1 H), 5.32-5.28 (dd, *J* = 8.5, 10.0 Hz,1 H), 5.08-5.06 (d, *J* = 8.5 Hz, 1 H), 3.57 (s, 3 H), 2.99 (s, 3 H) ppm; $^{13}$C NMR (125 MHz, $CDCl_3$): δ = 168.6, 147.2, 136.3, 134.6, 133.2, 132.8, 130.9, 129.2, 128.5, 128.1, 125.4, 61.8, 60.7, 58.3, 32.1 ppm.
